# Serum Troponin and Procedural Duration Predict Delayed iASD Closure After Left Atrial Arrhythmia Ablation

**DOI:** 10.3390/jcm15218357

**Published:** 2026-10-27

**Authors:** Ameen Abdulhasan Al-Alwany, Umniah Khajori, Mustafa Majid, Ghazi Farhan Haji, Giustino Varrassi

**Affiliations:** 1 College of Medicine, University of Baghdad, Bab Al-Muadham, Baghdad 10047, Iraq; omniya.rafed1700a@comed.uobaghdad.edu.iq (U.K.); mostafa.maged1600d@comed.uobaghdad.edu.iq (M.M.); ghazi_haji@comed.uobaghdad.edu.iq (G.F.H.); 2 Leipzig Heart Center, 04289 Leipzig, Germany; 3 Research Department, Fondazione Paolo Procacci, 00193 Roma, Italy; giuvarr@gmail.com

**Keywords:** iatrogenic atrial septal defect, transseptal puncture, radiofrequency ablation, cardiac troponin, left atrial tachyarrhythmia, atrial fibrillation, Wolff–Parkinson–White syndrome, myocardial injury

## Abstract

**Background:** Transseptal puncture is routinely performed to access the left atrium during catheter ablation of left atrial tachyarrhythmias. Although iatrogenic atrial septal defects (iASDs) frequently occur after the procedure, factors influencing delayed defect closure remain incompletely understood. Myocardial injury induced by radiofrequency (RF) ablation results in the release of cardiac troponin into the circulation and may reflect the extent of procedural tissue injury. **Methods:** This prospective study included patients who underwent RF ablation for left atrial arrhythmias at the Iraqi Center for Heart Diseases in collaboration with the College of medicine, University of Baghdad between March 2023 and April 2024. Serum troponin concentrations, procedural duration, and echocardiographic assessment of iASD size were evaluated prospectively to determine their association with delayed atrial septal defect closure over a period of 3–6 months. **Results:** Forty-seven patients were enrolled (mean age, 36.28 ± 10.34 years; 48.9% male). A significant positive association was observed between iASD size and serum troponin level (*p* < 0.001). For each unit increase in post-procedure troponin, the likelihood of ASD presence increases by more than two-fold at 3–6 months. Larger septal defects were also associated with longer procedural duration. **Conclusions:** Elevated serum troponin concentration following RF ablation is associated with larger iatrogenic atrial septal defects and may serve as a useful predictor of delayed iASD closure during the first six months of follow-up.

## 1. Introduction

Transseptal puncture (TSP) is an essential technique for obtaining access to the left atrium during catheter-based electrophysiological interventions. Although the procedure is routinely performed and generally considered safe, it inevitably creates an iatrogenic atrial septal defect (iASD) at the level of the fossa ovalis. The persistence of these defects following transseptal access has attracted increasing clinical interest because residual interatrial shunting may have important hemodynamic and clinical implications.

The reported incidence of persistent iASD varies considerably according to the ablation technique, sheath size, procedural characteristics, and duration of follow-up. In patients undergoing cryoballoon ablation using a 15-French sheath, persistent iASD has been reported in approximately 17–38% of patients after 4–6 months [1] and in 20–37% after 12–36 months [2,3]. In contrast, radiofrequency (RF) ablation performed with smaller 8–8.5-French sheaths is associated with lower reported incidences of approximately 2% at 4–6 months and 9–19% at 12–36 months, particularly in patients undergoing pulmonary vein isolation or ablation of left atrial tachyarrhythmias [3,4,5].

Despite its widespread use, TSP remains one of the most technically demanding components of left atrial catheter ablation. Although major complications such as cardiac perforation and cardiac tamponade are uncommon, they represent important procedural risks. In addition to these acute complications, the persistence of iASD has emerged as a relevant procedural consequence that may be influenced by multiple procedural and patient-related factors [5].

Structural remodeling of the left atrium is an important determinant of procedural outcome following catheter ablation. Restoration of sinus rhythm may promote reverse structural remodeling; however, the extent of recovery depends largely on the degree of pre-existing atrial fibrosis and the duration of exposure to arrhythmia-related stress [6]. Short-term stress primarily induces reversible ionic and genomic alterations, whereas prolonged exposure may result in irreversible cellular and extracellular matrix remodeling characterized by apoptosis and fibrosis [7]. Consequently, patients with relatively preserved atrial tissue may demonstrate greater structural recovery after successful ablation [5].

Embryologically, the interatrial septum is formed through fusion of the septum primum and septum secundum. The fossa ovalis represents the thin membranous remnant of the fetal foramen ovale and serves as the preferred site for transseptal puncture because of its anatomical characteristics. Mechanical dilation of this region during catheter manipulation may contribute to the formation and persistence of iatrogenic atrial septal defects [3,8,9,10].

Cardiac troponins are highly specific intracellular proteins involved in regulating myocardial contraction and are widely recognized as sensitive biomarkers of myocardial injury. Although initially established for diagnosing acute myocardial infarction, advances in assay technology have expanded their clinical utility to detecting myocardial injury associated with non-ischemic cardiac procedures [11]. Elevation of serum troponin concentrations has been consistently observed after several electrophysiological interventions, including electrical cardioversion, cardiac device implantation, and catheter ablation [12].

Radiofrequency catheter ablation intentionally creates localized myocardial necrosis to eliminate arrhythmogenic tissue. This controlled myocardial injury results in the release of intracellular troponin into the systemic circulation [13]. Previous prospective studies have demonstrated measurable post-procedural increases in cardiac troponin following RF ablation for supraventricular tachycardias, including atrioventricular nodal reentrant tachycardia, atrioventricular reentrant tachycardia, and atrial flutter. However, the clinical significance of post-ablation troponin elevation as a predictor of persistent iatrogenic atrial septal defects remains incompletely defined [14,15].

The present prospective study aimed to evaluate the association between serum troponin concentration, procedural duration, and the persistence of iatrogenic atrial septal defects following radiofrequency catheter ablation of left atrial arrhythmias.

## 2. Materials and Methods

### 2.1. Study Design and Patient Population

This prospective observational study was conducted at the Iraqi Center for Heart Diseases in collaboration with the College of medicine/University of Baghdad between March 2023 and April 2024. Patients undergoing radiofrequency (RF) catheter ablation for left atrial arrhythmias were consecutively enrolled to evaluate the association between serum troponin levels, procedural duration, and the persistence of iatrogenic atrial septal defects (iASDs) following transseptal puncture. Patients with recent acute coronary syndrome, prior septal manipulation and patients with heart failure were excluded from the study.

### 2.2. Troponin Measurement

Venous blood samples were collected 24 h after the ablation procedure and again three weeks later during follow-up. Serum troponin concentrations were measured using the AFIAS-6 automated fluorescent immunoassay system (Boditech Med Inc., Chuncheon, Republic of Korea). According to the manufacturer’s specifications, the 99th percentile upper reference limit in a healthy population is <0.03 ng/mL. The AFIAS-6 system quantitatively or semi-quantitatively measures the concentration of target analytes in blood, urine, and other biological samples using fluorescence immunoassay technology.

### 2.3. Transthoracic Echocardiographic Assessment

Transthoracic echocardiography was performed to evaluate the size of the iatrogenic atrial septal defect at 24 h and after 3–6 months. Measurements were obtained in the subcostal view using a GE Vivid E9 ultrasound system equipped with a 5 MHz phased-array transducer. Examinations were performed with patients in the supine and left lateral decubitus positions. Color Doppler imaging was used to determine both the size of the interatrial septal defect and the direction of interatrial shunt flow [16]. Ejection fraction, global longitudinal strain and left atrial volume index were measured for each patient.

### 2.4. Procedural Variables

Procedural characteristics were extracted from the electrophysiology procedure reports. The recorded variables included transseptal sheath size, number of sheaths used during the procedure, and total procedural duration. These parameters were subsequently analyzed for their association with iASD size and delayed defect closure.

### 2.5. Statistical Analysis

All collected data were entered into Microsoft Excel before statistical analysis using IBM SPSS Statistics for Windows (Version 23.0; IBM Corp., Chicago, IL, USA).

Continuous variables are presented as the mean ± standard deviation (SD), whereas categorical variables are expressed as frequencies and percentages. Descriptive and inferential statistical analyses were performed as appropriate.

Chi-square and Fisher’s exact tests were used for categorical associations; independent sample t tests were used for numerical variable comparisons.

Binary logistic regression analysis was used to identify independent predictors of atrial septal defect closure. Univariable models for each variable were separately used. Adjusted associations between ASD closure and the studied variables were expressed as odds ratios (ORs) with corresponding 95% confidence intervals (CIs). Receiver Operating Characteristic (ROC) curve analysis was performed to evaluate the diagnostic performance of post-procedure troponin in predicting persistent iASD at 3–6 months. The optimal decision threshold was identified by maximizing the Youden Index (*J* = Sensitivity + Specificity − 1).

All statistical tests were two-sided, and a *p*-value < 0.05 was considered statistically significant.

## 3. Results

### 3.1. Baseline Characteristics

A total of 47 patients undergoing radiofrequency catheter ablation for left atrial supraventricular arrhythmias were included in the study. The study population comprised 23 males (48.9%) and 24 females (51.1%), with an overall mean age of 36.28 ± 10.34 years. The mean age was 35.13 ± 8.93 years in males and 37.38 ± 11.62 years in females. The mean body surface area (BSA) for the entire cohort was 1.86 ± 0.16 m^2^. Baseline demographic characteristics are summarized in Table 1. The mean BMI of participants was 26.65 ± 2.6. The mean (SD) values for echocardiography variables were as follows: GLS = −18.62 (2.86), EF = 64.68 (5.23); LAVI = 27.94 (5.08).

Among the enrolled patients, 38 (80.9%) underwent radiofrequency ablation for Wolff–Parkinson–White (WPW) syndrome, whereas 9 (19.1%) underwent pulmonary vein isolation for atrial fibrillation (AF). All procedures were performed using a single transseptal puncture. Distribution according to sheath size and catheter approach is presented in Table 2.

### 3.2. Procedural Characteristics

Procedural duration differed substantially according to the underlying arrhythmia. Patients undergoing pulmonary vein isolation for atrial fibrillation required significantly longer procedures, with a mean procedural ablation time of 97.0 ± 28.1 min, whereas left-sided accessory pathway ablation for WPW syndrome required a mean procedural ablation time of only 3.49 ± 2.48 min.

### 3.3. Association Between Arrhythmia Type, Sheath Size, and iASD Size

At the initial follow-up evaluation, iASD size demonstrated a significant association with the underlying arrhythmia type (*p* < 0.0001). Larger atrial septal defects were observed predominantly in patients undergoing pulmonary vein isolation for atrial fibrillation, whereas patients with WPW syndrome generally exhibited smaller defects (Table 3).

A significant relationship was also identified between transseptal sheath size and iASD size (*p* < 0.0001). Procedures performed using an 8-F sheath were associated with smaller atrial septal defects, whereas larger defects were more frequently observed following procedures performed with a 12-F sheath or with combined 8-F and 12-F sheath use (Table 3).

### 3.4. Association Between iASD Size and Procedural Duration

Procedural duration increased significantly with increasing iASD size (*p* < 0.0001). Patients with a 2 mm defect had a mean procedural duration of 2.97 ± 0.78 min, whereas patients with a 5 mm defect had a mean procedural duration of 112.50 ± 13.89 min. Intermediate procedural durations were observed in patients with 3 mm and 4 mm defects (Table 4).

### 3.5. Association Between iASD Size and Serum Troponin Concentration

Serum troponin concentrations measured after the procedure were significantly associated with iASD size (*p* < 0.0001). Patients with a 2 mm atrial septal defect demonstrated a mean serum troponin concentration of 1.77 ± 0.92 ng/mL, whereas those with a 5 mm defect demonstrated substantially higher concentrations (7.39 ± 0.70 ng/mL). Progressive increases in troponin levels were observed with increasing defect size (Table 5).

In ordinal regression, specifically, for each unit increase in post-procedure troponin, the odds of falling into a higher iASD size category (2–5 mm) increased more than three-fold (OR = 3.414, 95% CI: 1.887–6.178).

### 3.6. Association Between Sheath Configuration and Serum Troponin Level

Serum troponin concentrations differed significantly according to catheter configuration. Procedures performed using a double-catheter approach demonstrated significantly higher post-procedural troponin concentrations than those performed using a single-catheter approach (*p* < 0.0001), indicating greater myocardial injury associated with more complex transseptal instrumentation (Table 6).

### 3.7. Follow-Up Troponin Measurements

Troponin concentrations were evaluated at 24 h after ablation and again three weeks later. Although serum troponin levels had returned to values close to the normal range at follow-up, significant associations remained between the initial post-procedural troponin concentration and iASD size, procedural duration, and arrhythmia type (all *p* < 0.0001). Patients with larger atrial septal defects (4–5 mm), procedures lasting more than 10 min, and atrial fibrillation demonstrated significantly higher initial troponin concentrations than their respective comparison groups (Table 7).

All iASDs were closed at 6 months and 74.5% of all of them were closed after 3 months.

Binary logistic regression demonstrated that higher post-procedure troponin significantly increases the odds of ASD presence at 3–6 months (*B* = 0.769, Wald = 12.454, *p* < 0.001). For each unit increase in post-procedure troponin, the likelihood of ASD presence increases by more than two-fold (OR = 2.158, 95% CI: 1.408 − 3.308).

To account for potential confounding by procedure type, using Firth penalized logistic regression, troponin was independently associated with ASD persistence at 3–6 months (OR 1.82, 95% CI 1.14–4.94; *p* = 0.018). Procedure type was not significantly associated with ASD persistence (OR 1.47, 95% CI 0.01–20.39; *p* = 0.810). Interpretation may be limited by the small number of patients with AF.

ROC curve analysis demonstrated high overall accuracy for post-procedure troponin in predicting residual ASD presence at 3–6 months, with an Area Under the Curve (AUC) of 0.85, *p* < 0.001. The optimal cut-off value was determined to be 3.15 ng/mL (Youden Index = 0.66), yielding a sensitivity of 75.0% and a specificity of 91.4%.

### 3.8. Other Clinical and Echocardiographic Factors

Using binary logistic regression, the following predictions were made on the presence of iASD at 3–6 months:

1. Age and body mass index: Both show statistically insignificant prediction (OR = 1.072, 1.22, respectively. *p*-value: 0.05, 0.118, respectively).
2. Ejection fraction and Longitudinal strain (GLS): Statistically significant inverse association between baseline ejection fraction and residual ASD presence at 3–6 months (*B* = −0.211, Wald = 5.781, *p* = 0.016; OR = 0.81). On the other hand, GLS did not significantly predict iASD (*p*-value of 0.152, OR = 1.2).
3. Left atrial volume index (LAVI): Higher LAVI was significantly associated with ASD persistence at 3–6 months (OR = 1.27, 95% CI: 1.06–1.52, *p* = 0.010).

## 4. Discussion

The present prospective study investigated the relationship between serum troponin concentration, procedural duration, and the persistence of iatrogenic atrial septal defects (iASDs) following transseptal puncture for radiofrequency catheter ablation of left atrial arrhythmias. The principal findings demonstrate that larger iASDs are associated with higher post-procedural serum troponin levels and longer procedural duration, suggesting that both biomarkers may reflect the extent of procedural myocardial and septal injury [3,16].

Transseptal catheterization has become an indispensable component of modern electrophysiological interventions for left-sided supraventricular arrhythmias. Although the formation of an iatrogenic atrial septal defect is an inevitable consequence of transseptal puncture rather than a procedural complication, the persistence of these defects remains clinically relevant because of the potential for residual interatrial shunting. Despite the widespread use of this technique, the natural history of iASD closure and the factors influencing delayed healing remain incompletely understood.

In the present study, spontaneous closure of the iASD occurred in 35 of 47 patients (74%), whereas persistent defects remained in 12 patients (26%) during follow-up. This persistence rate is higher than that reported by Rillig et al. [15], who observed spontaneous closure in 96.3% of patients three months after pulmonary vein isolation. Similarly, Obel et al. [16,17] reported an incidence of persistent iASD of only 6.5% after a longer follow-up period, while Anselmino et al. [17] documented a persistence rate of 5.6% twelve months after radiofrequency ablation. The variability among these studies is likely to be attributable to differences in patient selection, procedural techniques, sheath size, follow-up duration, and imaging protocols.

Our findings further demonstrate that atrial fibrillation procedures requiring pulmonary vein isolation were associated with significantly larger iASDs than procedures performed for Wolff–Parkinson–White syndrome. Likewise, larger defects were observed when larger transseptal sheaths or dual-catheter techniques were used. These observations support previous reports indicating that extensive catheter manipulation across the interatrial septum contributes to persistent septal injury. Pulmonary vein isolation commonly requires the use of larger sheaths (8–12 Fr), repeated catheter manipulation, and prolonged transseptal instrumentation, all of which may increase mechanical stress on the fossa ovalis and delay spontaneous defect closure [16].

Our results are consistent with those reported by Rillig et al. [15], who highlighted the relatively high prevalence of persistent iASD after cryoballoon pulmonary vein isolation and after single transseptal puncture using a double-catheter approach. Conversely, Nagy et al. [18] suggested that the persistence of iASD is relatively independent of the ablation technique itself. In contrast, Fitchet et al. [19] reported no persistent atrial septal defects after ablation of left-sided accessory pathways using a single-ablation catheter. The discrepancy between these findings may reflect differences in procedural complexity. Compared with accessory pathway ablation, pulmonary vein isolation requires substantially greater catheter manipulation and prolonged mechanical stress at the puncture site, thereby increasing the likelihood of persistent septal defects [16].

Procedural duration was another important determinant identified in the present study. Patients undergoing pulmonary vein isolation required significantly longer procedures than those undergoing ablation for accessory pathways, and increasing procedural duration was strongly associated with larger iASDs. These findings agree with previous studies demonstrating that prolonged transseptal instrumentation and repeated catheter manipulation are important contributors to persistent atrial septal defects. Hammerstingl et al. [20] similarly reported that pulmonary vein isolation procedures are substantially longer than other transseptal interventions and suggested that prolonged catheter manipulation contributes to delayed septal healing. Obel et al. [16] also identified prolonged transseptal instrumentation as an important procedural risk factor for persistent iASD [20,21,22,23,24].

An important finding of the present study is the significant association between serum troponin concentration and iASD size. Radiofrequency ablation intentionally produces localized myocardial necrosis to eliminate arrhythmogenic tissue, resulting in the release of intracellular cardiac troponin into the systemic circulation. Accordingly, greater procedural injury would be expected to produce higher circulating troponin concentrations. Our results demonstrate a progressive increase in serum troponin levels with increasing defect size, suggesting that post-procedural troponin may serve as a surrogate marker of the extent of procedural myocardial and septal injury [25].

These findings are consistent with previous investigations demonstrating significant elevations in cardiac troponin following radiofrequency catheter ablation. Earlier studies evaluating myocardial injury after electrophysiological procedures similarly concluded that serum troponin reflects the degree of procedural myocardial damage [25,26,27,28]. Because the left atrium contains abundant cardiomyocytes expressing intracellular troponin, radiofrequency-induced myocardial necrosis is expected to result in measurable systemic release of this biomarker [26,27]. The present findings therefore support the potential clinical utility of post-ablation serum troponin as an indicator of procedural tissue injury and as a predictor of delayed iASD closure [29,30,31,32].

## 5. Study Limitations

This study has several limitations that should be considered when interpreting the findings. First, the study was conducted at a single tertiary referral center with a relatively modest sample size, limiting regression utility, which may limit the generalizability of the results to broader patient populations. Second, the follow-up period was limited to the assessment of atrial septal defect closure during the first six months after radiofrequency catheter ablation; therefore, the long-term clinical significance of persistent iatrogenic atrial septal defects could not be evaluated. Third, although significant associations between serum troponin concentration, procedural duration, and iASD size were demonstrated, causality cannot be established because of the observational nature of the study. Finally, the study evaluated serum troponin as a marker of procedural myocardial injury but did not include additional biomarkers or advanced imaging modalities that might further characterize tissue injury and atrial remodeling.

Despite these limitations, the prospective design, standardized assessment of serum troponin, and echocardiographic evaluation of iatrogenic atrial septal defects provide clinically relevant evidence supporting the role of post-procedural troponin concentration as a potential predictor of delayed iASD closure.

## 6. Conclusions

Radiofrequency catheter ablation of left atrial arrhythmias results in measurable myocardial injury that is reflected by increased post-procedural serum troponin concentrations. In the present study, elevated serum troponin levels were significantly associated with larger iatrogenic atrial septal defects and prolonged procedural duration. Patients undergoing more complex procedures, particularly pulmonary vein isolation, demonstrated larger septal defects and higher troponin concentrations than those undergoing ablation for left-sided accessory pathways.

These findings suggest that serum troponin may serve as a useful biomarker for identifying patients at increased risk of large iatrogenic atrial septal defect closure. Further multicenter studies with larger patient populations and longer follow-up are warranted to validate these observations and to determine their clinical implications.

## Figures and Tables

**Table 1:** Baseline demographic and clinical characteristics of the study population (n = 47).

Variable	Male (*n* = 23)	Female (*n* = 24)	Total (*n* = 47)
Age (years), mean ± SD	35.13 ± 8.93	37.38 ± 11.62	36.28 ± 10.34
Age range (years)	17–53	19–57	17–57
Body surface area (m^2^), mean ± SD	1.91 ± 0.16	1.80 ± 0.15	1.86 ± 0.16
Body surface area range (m^2^)	1.60–2.20	1.60–2.10	1.60–2.20

Values are presented as mean ± standard deviation (SD) or range.

**Table 2:** Distribution of arrhythmia type according to transseptal sheath configuration.

Arrhythmia	8-F Sheath	12-F Sheath	Combined 8-F + 12-F	Total
WPW	24 (63.2%)	14 (36.8%)	0	38
AF	0	3 (33.3%)	6 (66.7%)	9
Total	24 (51.1%)	17 (36.2%)	6 (12.8%)	47

AF, atrial fibrillation; WPW, Wolff–Parkinson–White syndrome.

**Table 3:** The Relationship between transseptal sheath size and iASD size.

(A)					
Arrhythmia	2 mm	3 mm	4 mm	5 mm	*p*-Value
WPW	6	21	11	0	
AF	0	0	1	8	<0.0001
**(B)**					
**Sheath Size**	**2 mm**	**3 mm**	**4 mm**	**5 mm**	***p*** **-Value**
8 F	6	18	0	0	
12 F	0	3	12	2	
8 F + 12 F	0	0	0	6	<0.0001

**Table 4:** Association between iatrogenic atrial septal defect size and procedural duration.

ASD Size	n	Procedure Duration (min), Mean ± SD	Range	*p*-Value
2 mm	6	2.97 ± 0.78	2.00–4.33	
3 mm	21	3.04 ± 0.52	2.00–4.00	
4 mm	12	13.75 ± 14.71	6.60–90.00	
5 mm	8	112.50 ± 13.89	90.00–120.00	<0.0001

**Table 5:** Association between iatrogenic atrial septal defect size and serum troponin concentration.

ASD Size	n	Troponin (ng/mL), Mean ± SD	Range	*p*-Value
2 mm	6	1.77 ± 0.92	0.90–3.20	
3 mm	21	1.83 ± 0.57	0.75–3.20	
4 mm	12	2.63 ± 1.67	1.20–7.60	
5 mm	8	7.39 ± 0.70	6.40–8.20	<0.0001

**Table 6:** Serum troponin concentration according to transseptal sheath configuration.

Procedure	Troponin (ng/mL), Mean ± SD	*p*-Value
Single 8-F sheath	1.76 ± 0.66	
Single 12-F sheath	3.18 ± 2.29	
Double sheath	7.18 ± 0.69	<0.0001

**Table 7:** (**A**): Association between serum troponin concentration and iASD size. (**B**): Association between serum troponin concentration and procedural duration. (**C**): Association between serum troponin concentration and arrhythmia type.

(A)				
ASD Size	n	Troponin 24 h	Troponin 3 Weeks	*p*-Value
2–3 mm	27	1.81 ± 0.64	0.015 ± 0.007	<0.0001
4–5 mm	20	4.53 ± 2.74	0.021 ± 0.009	<0.0001
**(B)**				
**Procedure Duration**	**n**	**Troponin 24 h**	**Troponin 3 Weeks**	***p*** **-Value**
≤10 min	37	1.945 ± 0.630	0.015 ± 0.007	<0.0001
>10 min	10	6.76 ± 2.15	0.027 ± 0.007	<0.0001
**(C)**				
**Arrhythmia**	**n**	**Troponin 24 h**	**Troponin 3 Weeks**	***p*** **-Value**
WPW	38	2.06 ± 1.15	0.015 ± 0.007	<0.0001
AF	9	6.80 ± 1.81	0.028 ± 0.004	<0.0001

## Data Availability

The data supporting the findings of this study are available from the corresponding author upon reasonable request.

## Abbreviations

AF	Atrial Fibrillation
ASD	Atrial Septal Defect
iASD	Iatrogenic Atrial Septal Defect
TSP	Transseptal Puncture
RF	Radiofrequency
PVI	Pulmonary Vein Isolation
WPW	Wolff–Parkinson–White Syndrome
SVT	Supraventricular Tachycardia
LA	Left Atrium
BSA	Body Surface Area
